# Computed tomography urography with corticomedullary phase can exclude urinary bladder cancer with high accuracy

**DOI:** 10.1186/s12894-022-01009-4

**Published:** 2022-04-12

**Authors:** Suleiman Abuhasanein, Carl Hansen, Dragan Vojinovic, Staffan Jahnson, Henrik Leonhardt, Henrik Kjölhede

**Affiliations:** 1 Department of Urology, Institute of Clinical Science, Sahlgrenska Academy, University of Gothenburg, 413 90 Göteborg, Sweden; 2 Department of Surgery, Urology Section, NU Hospital Group, Uddevalla, Region Västra Götaland, Sweden; 3 Department of Radiology, NU Hospital Group, Uddevalla, Region Västra Götaland, Sweden; 4 Department of Clinical and Experimental Medicine, Division of Urology, Linköping University, Linköping, Sweden; 5 Department of Radiology, Institute of Clinical Science, Sahlgrenska Academy, University of Gothenburg, Göteborg, Sweden; 6 Department of Radiology, Sahlgrenska University Hospital, Göteborg, Sweden; 7 Department of Urology, Sahlgrenska University Hospital, Region Västra Götaland, Göteborg, Sweden

**Keywords:** Bladder cancer, Computed tomography (CT), Diagnostic accuracy, Early detection of cancer, Hematuria, Urography

## Abstract

**Background:**

To evaluate the diagnostic accuracy of computed tomography-urography (CTU) to rule out urinary bladder cancer (UBC) and whether patients thereby could omit cystoscopy.

**Methods:**

All patients evaluated for macroscopic hematuria with CTU with cortico-medullary phase (CMP) and cystoscopy at our institute between 1^st^ November 2016 and 31^st^ December 2019 were included. From this study cohort a study group consisting of all UBC patients and a control group of 113 patients randomly selected from all patients in the study cohort without UBC. Two radiologists blinded to all clinical data reviewed the CTUs independently. CTUs were categorized as positive, negative or indeterminate. Diagnostic accuracy and proportion of potential omittable cystoscopies were calculated for the study cohort by generalizing the results from the study group.

**Results:**

The study cohort consisted of 2195 patients, 297 of which were in the study group (UBC group, n = 207 and control group, n = 90). Inter-rater reliability was high (κ 0.84). Evaluation of CTUs showed that 174 patients were assesessed as positive (showing UBC), 46 patients as indeterminate (not showing UBC but with limited quality of CTU), and 77 patients as negative (not showing UBC with good quality of CTU). False negative rate was 0.07 (95%, CI 0.04–0.12), false positive rate was 0.01 (95% CI 0.0–0.07) and negative predictive value was 0.99 (95% CI 0.92–1.0). The area under the curve was 0.93 (95% CI 0.90–0.96). Only 2.9% (3/102) with high-risk tumors and 11% (12/105) with low- or intermediate-risk tumors had a false negative CTU. Cystoscopy could potentially have been omitted in 57% (1260/2195) of all evaluations.

**Conclusions:**

CTU with CMP can exclude UBC with high accuracy. In case of negative CTU, it might be reasonable to omit cystoscopy, but future confirmative studies with possibly refined technique are needed.

**Supplementary Information:**

The online version contains supplementary material available at 10.1186/s12894-022-01009-4.

## Background

Urinary bladder cancer (UBC) is the 10^th^ most common diagnosed cancer in the world in 2020 [1]. The most common sign of UBC is macroscopic hematuria, which is investigated by cystoscopy and computed tomography-urography (CTU) [2, 3]. Cystoscopy currently cannot be replaced by cytology or by any other non-invasive test, while CTU can be performed only in certain cases, e.g., multiple or high-risk tumors [2].

Since cystoscopy is considered the reference examination for diagnosis of UBC, the bladder is often largely ignored by radiologists during routine CTU interpretation [4]. CTU is defined by the European Society of Urogenital Radiology (ESUR) as a multiphasic imaging modality for the urinary tract including the urinary bladder and using an intravenous administration of contrast medium [5, 6].

Several CTU techniques, using one or multiple phases, single- or split bolus intravenous injection of contrast medium have been introduced [6]. Traditionally, CTU consists of an unenhanced phase (UP), a nephrographic phase (NP), and a mandatory excretory phase (EP) [3, 7, 8]. CTU with these three phases has been demonstrated to have a high specificity for UBC, which allows patients with positive findings to go directly to transurethral resection of bladder (TURBT) without a preceding cystoscopy [3]. However, most patients still undergo cystoscopy, which is an invasive examination with patient discomfort and with risk for complications such as hematuria, infection and voiding problems [9–11].

UBCs have a detectable early enhancement of contrast media, which may be used to detect UBC rather than only as filling defects in EP [12]. Therefore, it has been proposed that CTU including a corticomedullary phase (CMP), i.e. an arterial phase, could be comparable to cystoscopy in the detection of UBC with a higher sensitivity than CTU without CMP [13]. CTU followed by cystoscopy, according to the Swedish standardized care pathway (SCP), has been the clinical routine in our institute for evaluation of macroscopic hematuria, and since 2016 included CMP.

## Methods

The aim of this study was to determine the diagnostic accuracy, especially the false negative rate (FNR), of CTU with CMP in exclusion of UBC and whether some patients could omit cystoscopy.

### Patients

**Fig. 1:**
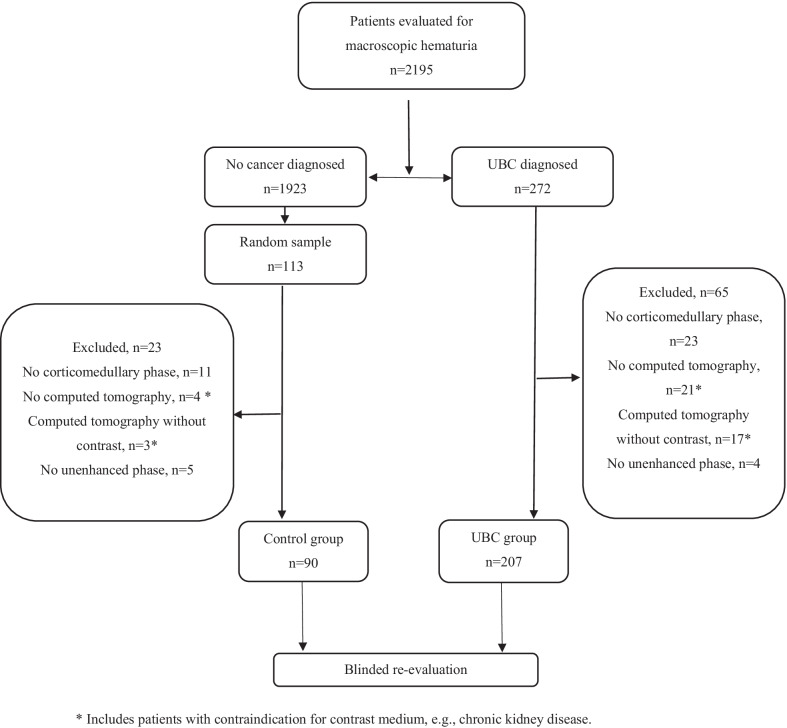
STARD flow diagram showing inclusion of patients in the study for blinded evaluation of computed tomography urography with cortico-medullary phase. STARD: Standards for the Reporting of Diagnostic Accuracy Studies; UBC: Urinary bladder cancer

All patients evaluated for macroscopic hematuria according to SCP in the NU Hospital Group, Uddevalla, Sweden, between 1^st^ November 2016 and 31^st^ December 2019 were retrospectively included. The criteria for SCP, and therefore the inclusion criteria of the study, were macroscopic hematuria and ≥ 40 years age. The age criterion was changed to ≥ 50 years in 2018. Patients were excluded from the study if they did not undergo CTU, or if the CTU was not done with CMP or UP (Fig. 1).

The study group was divided into the UBC group, which consisted of all patients diagnosed with UBC during the study period, while a random sample of patients who had macroscopic hematuria but without detected UBC, matched by year of diagnosis, constituted the control group. From this study group the results were generalized to the study cohort.

Medical records were retrospectively reviewed to retrieve tumor-specific data for the UBC group (e.g., tumor number and tumor size which were estimated during TURBT using the loop of the resectoscope (7 mm as a reference) and to ensure that patients in the control group were not diagnosed with UBC until 31st March 2021. All UBC patients underwent TURBT and had a histopathologically verified UBC diagnosis.

### Imaging technology

Preparation before examination included drinking of 1000 mL of water and not voiding approximately 90 min prior to examination. CTU was performed with the patient in supine position, using a 64-detector scanner (General Electric, Boston, USA or Siemens Medical solutions, Forchheim, Germany). A four-phase protocol including UP of the abdomen and pelvis (70–480 mA) was initiated. This was followed by a CMP of the abdomen and pelvis (120–560 mA) at bolus tracking + 20 s after intravenously administration of iodinated contrast medium (Iohexol 350 mg/mL; 400 mg I/kg, 20 g I/kg/sec, Omnipaque; GE Healthcare, Waukesha, WI, USA).

The following phase was a nephrographic phase (NP) of the kidneys (120–560 mA) at bolus tracking + 40 s. After a short mobilization of the patient, an excretory phase (EP) of the abdomen and pelvis (70–480 mA) > 7.5 min after contrast medium administration was obtained. All four phases were reduced with 40% mAs with GE or made with a quality reference mAs Siemens. In other words, the UP and EP could be considered as “low-dose” phases and the CMP as a “normal-dose” phase concerning radiation. Collimation of 0.6 mm, pitch of 1.4 and 120 kVp were applied in all phases. No diuretic drugs were given.

### Imaging analysis

All included CTU scans were copied to a separate study database, pseudonymized and purged of all annotations. Two specialists in radiology (CH and DV) with seven and eighteen years of experience of interpretation of CTU who were blinded to all clinical data and previous CTU results, reviewed the pseudonymized CTUs independently. The number of tumors and the largest size of the largest tumor were recorded. In addition, inadequacy of bladder filling volume, existence of bladder stones, indwelling catheter, existence of thickened bladder wall and other image distortions that made the re-evaluation difficult, such as artifacts due to hip prostheses, were also noted separately.

The reviewers categorized the CTUs as either positive (showing UBC) or negative (not showing UBC). The negative CTUs were then further categorized as having good image quality (good/very good bladder filling, no non-specific bladder wall thickening, no indwelling catheter, no bladder stones, and no significant image distortions) or having limited image quality (having one or more of the above). The CTUs were thereby categorized as positive (POS), indeterminate (not showing UBC but with limited quality, IND), or negative (not showing UBC with good quality, NEG). After the first round of review, a consensus between the two radiologists was reached in a joint review in the cases where the interpretations differed. All statistical analyses were based on this consensus interpretation if not otherwise indicated. After unblinding, one further round of review of the cases with false negative CTU in the group (NEG) was performed by a senior radiologist (HL), who was not blinded to the results of the other radiologists interpretations to try to identify possible systematic errors in interpretation.

### Statistical analysis

All available UBC patients were included in the study. To be able to demonstrate an expected FNR of around 10% and a false positive rate (FPR) < 50% the required sample size was calculated to approximately 200 UBC patients and 100 controls [14]. The control group was increased by approximately 10% to account for exclusions and were randomly selected from the patients without UBC, matched by year of diagnosis, for a total of 113 patients. This sampling of the controls was done to keep the number of re-evaluations manageable and focused on the FNR, while still being able to estimate other accuracy measures and omittable cystoscopies. This procedure allowed us to generalize the results from the study group to the study cohort with highly valid results.

Descriptive statistics were used for patient characteristics (age at CTU and sex) and tumor characteristics (clinical tumor stage, tumor size and number of tumors). Continuous data were presented as mean with standard deviation (SD). Inter-rater variability of the interpretations of the CTUs was analyzed with Cohen’s kappa with > 0.80 interpreted as good agreement. Receiver operating characteristic analysis was done and the area under the curve (AUC) was calculated.

The FNR and FPR were calculated with 95%-confidence intervals (CI) according to the score method with continuity correction described by Wilson [15]. Positive predictive value (PPV) and negative predictive value (NPV) were calculated with 95% CI similarly, and were based on the entire cohort of patients with macroscopic hematuria, i.e., results of the control group were multiplied to represent the entire cohort without UBC, assuming similar results in randomly selected controls and in those patients without UBC who were not evaluated in the present study. Statistical analysis was performed using SPSS version 27 (IBM Corp., Armonk, NY, USA).

Omittable cystoscopies was defined as the POS CTUs, where the patient could go directly to a TURBT, and the NEG CTUs. This was calculated as a proportion of the entire cohort of patients with macroscopic hematuria, i.e., the randomly selected control group was generalized to reflect the entire group of patients without UBC, and the patients that had not undergone a CTU according to the protocol were counted as requiring cystoscopy. The number of cystoscopies needed to detect one case of UBC, and one case of high-risk UBC, respectively, among the NEG CTUs were calculated.

## Results

### Patients

We identified 2195 patients who had been investigated for macroscopic hematuria in our institute in the study period. Of these, 272 (12%) patients were diagnosed with UBC. Of the remaining 1923 patients without UBC, 113 (6%) were randomly selected for the control group. After exclusion of 65 (24%) patients in the UBC group and 23 (20%) patients in the control group, the final re-evaluation cohort consisted of 297 patients; 207 with UBC and 90 controls (Fig. 1, Additional file 1: Table 1). The characteristics of the included patients are detailed in Table 1. There were no cases of primary carcinoma in-situ.

**Table 1:** Descriptive parameters of all patients included in the study

Variable name		All	Control group	Cancer group
No.patients		297	90	207
Sex	Male	207 (70)	56 (62)	151 (73)
Age	Mean (SD)	72 (11)	68 (12)	74 (9)
CTU	Four phases	290 (98)	88 (98)	202 (98)
Filling volume	Good/very good	285 (96)	88 (98)	197 (95)
Indwelling catheter		26 [9]	8 [9]	18 [9]
Bladder stones		13 [4]	8 [9]	5 [2]
Thick bladder wall		38 [13]	8 [9]	30 [14]
Other image distortions		69 (23)	12 [13]	57 (28)
General quality of CTU	Good/very good	283 (95)	88 (98)	195 (94)
Solitary tumor*			–	133 (66)
Size of tumor**	0–10 mm		–	23 [11]
	11–30 mm		–	99 (48)
	> 30 mm		–	52 (25)
Local tumor stage***	cTaG1		–	22 [11]
	cTaG2		–	83 (40)
	cTaG3		–	10 [5]
	cT1G1		–	1 (0.5)
	cT1G2		–	20 (9.5)
	cT1G3		–	31 [15]
	cT2		–	40 [19]
Concomitant Cis****	cTaG3 + Cis		–	4 (33)
	cT1 + Cis		–	5 (42)
	cT2 + Cis		–	3 (25)
Lymph node metastasis			–	5 [2]
Distant metastasis			–	2 [1]

Figures represent number of patients (% of numbers of the column) if not otherwise indicated

Cis: Carcinoma in situ; CTU: Computed tomography-urography; SD: Standard deviation

*According to TURBT reports

**Missing cases 33

***Corrected after second look resection if appropriate

****There was no primary cTis

In 19% (40/207) of the UBC patients, the previous clinical CTU reports described a clear/suspect UBC, leading the patient directly to TURBT without a preceding cystoscopy. In a further two patients where CTU showed UBC, outpatient cystoscopy was not possible due to urethral strictures and thereby diagnosed at cystoscopy in anesthesia and TURBT was performed at the same time.

**Fig. 2:**
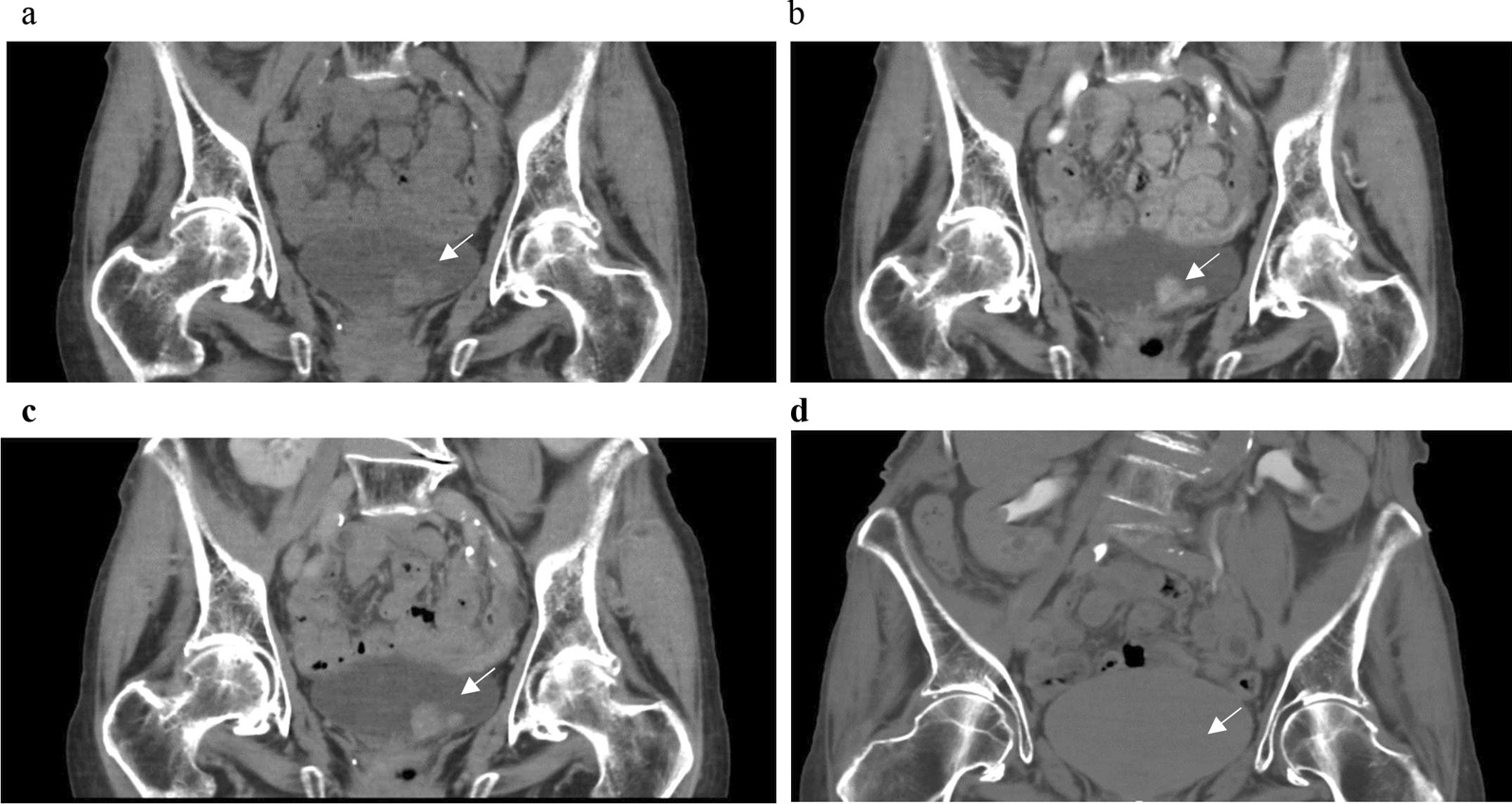
Urinary bladder cancer attenuation in a coronal computed tomography urography reconstruction in: **a** unenhanced phase, **b** corticomedullary phase (CMP), **c** nephrographic phase, and **d** excretory phase. Notice that the tumor is clearly seen in CMP due to good enhancement and it is hidden in the not optimal contrast-mixed urine in the bladder in (**d**), sometimes happening despite efforts to get high concentration and volume of contrast in the bladder

Of the remaining 165 UBC patients who underwent outpatient cystoscopy, UBC was missed in ( 4/165, 2.4%) which were diagnosed later due to either malignant cells in urine cytology (n = 1), at surgery for bladder stones (n = 1), or after histopathological result of a biopsy from suspected cystitis (n = 1). One UBC was missed at cystoscopy that was interpreted as cystitis cystisca but the CTU indicated UBC, which led to a TURBT. An example of a CTU in different contrast phases from a patient with UBC is shown in Fig. 2.

### CTU interpretations

Reviewer 1 classified 171 (58%) as POS, 79 (27%) as NEG, and 47 (16%) as IND, while reviewer 2 classified, 154 (52%) as POS, 82 (28%) as NEG, and 61 (21%) as IND. Cohen’s kappa (POS vs NEG or IND) was 0.84 (95% CI 0.78–0.91; *p* < 0.001) indicating a good inter-observer agreement. The reviewers differed in interpretation in 23 (8%) cases, for which consensus was reached after a second round of review (Table 2). For the consensus interpretations, the FNR was 0.07 (95% CI 0.04–0.12) while FPR was 0.01 (95% CI 0.00–0.07). AUC was 0.93 (95% CI 0.90–0.96). PPV was 0.91 (95% CI 0.86–0.94) and NPV was 0.98 (95% CI 0.97–0.98).

**Table 2:** Diagnostic accuracy of CTU for the detection of bladder cancer from both reviewers and their consensus

	Consensus		Reviewer 1		Reviewer 2	
	Cancer	Control	Cancer	Control	Cancer	Control
Positive, n	173	1	170	1	154	0
Indeterminate, n	19	27	20	27	33	28
Negative, n	15	62	17	62	20	62
FNR	0.07 (0.04–0.12)		0.08 (0.05–0.13)		0.10 (0.06–0.15)	
FPR	0.01 (0.00–0.07)		0.01 (0.00–0.07)		0.00 (0.00–0.05)	
NPV*	0.99 (0.92–1.00)		0.98 (0.92–1.00)		0.98 (0.92–1.00)	
PPV*	0.91 (0.86–0.95)		0.91 (0.86–0.95)		1.00 (0.97–1.00)	

Confidence intervals (95%) in parentheses

CTU: Computed tomography-urography; FNR: False negative rate; FPR: False positive rate; PPV: Positive predictive value; NPV: Negative predictive value

*Values were calculated for the entire cohort of 2195 patients with a cancer prevalence of 12% and after matched exclusions

### False negative CTUs

**Fig. 3:**
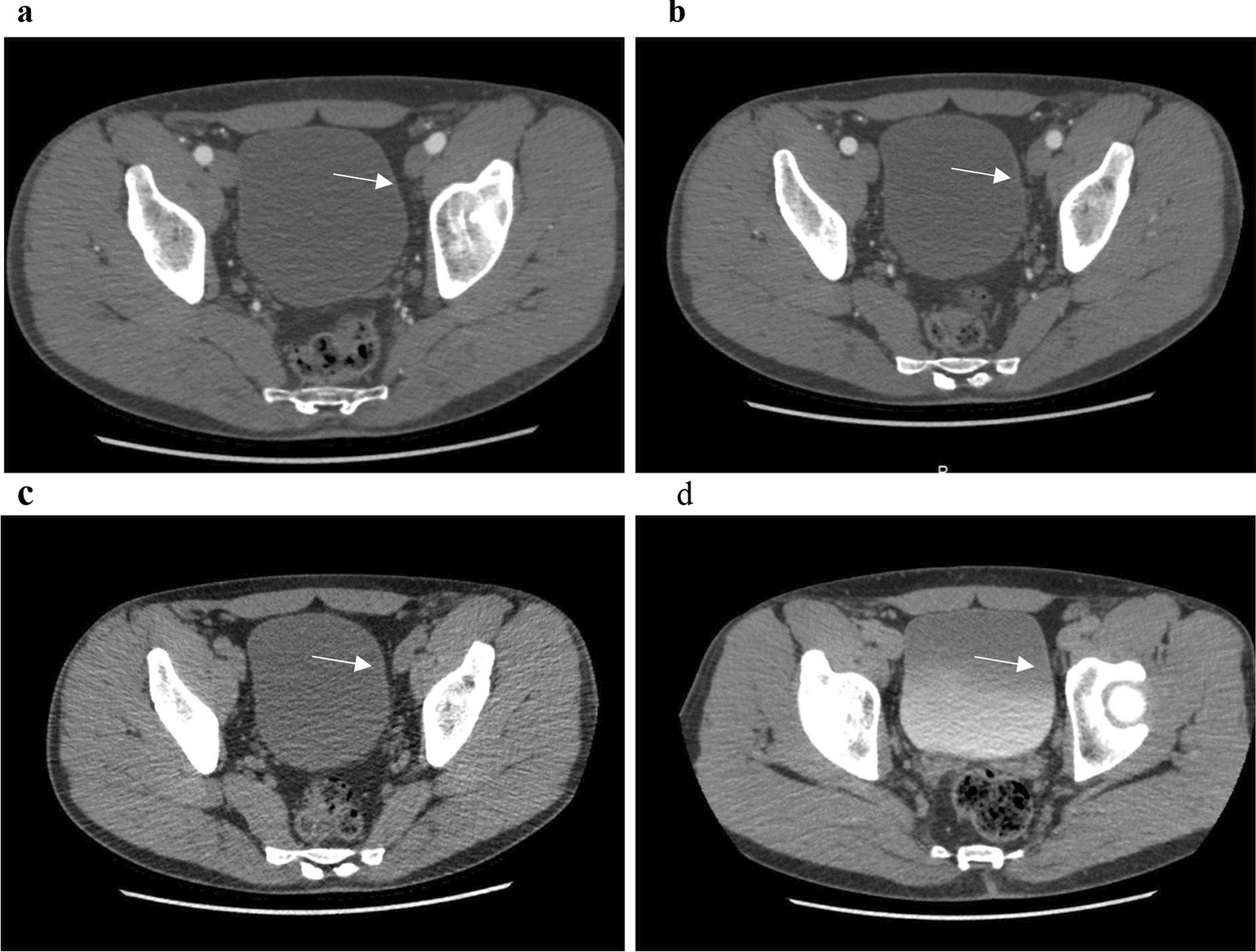
A flat (sessile) 3 cm T1G2 tumor in the urinary bladder which was missed in a computed tomography urography with high quality. An axial reconstruction in: **a** unenhanced phase, **b** corticomedullary phase (CMP), **c** nephrographic phase, and **d** excretory phase

The characteristics of the patients with tumors that were not identified on CTU, grouped by the consensus interpretations, are detailed in Table 3. Among the fifteen patients with NEG CTUs, only three patients (4% of NEG CTUs, 2.9% of high-risk tumors, and 1.4% of all tumors) had high-risk tumors (TaG3, T1, or T2), while the remaining twelve patients (16% of NEG CTUs, 11% of low/intermediate-risk tumors and 5.7% of all tumors) had low-grade Ta tumors. These fifteen cases with NEG CTU were re-evaluated again by a senior radiologist (HL) and all were interpreted again as negative. There were four patients with tumors > 10 mm in the largest diameter which were classified as NEG CTU. These were all flat and with no significant contrast enhancement (Fig. 3).

**Table 3:** Descriptive parameters of the patients with missed tumors grouped by the consensus interpretations (indeterminate or negative CTUs)

		Indeterminate	Negative
No.patients		19	15
Age (years)	Mean (SD)	79 [8]	69 [13]
Male sex		15 (79)	10 (67)
Solitary tumor		9 (50)*	10 (67)
Size of largest tumor**	0–10 mm	2 [13]	8 (67)
	11–30 mm	10 (62)	3 (25)
	> 30 mm	4 (25)	1 [8]
Local tumor stage	cTaG1	4 [21]	6 (40)
	cTaG2	6 (31)	6 (40)
	cTaG3	3 [16]	0 (0)
	cT1G1	0 (0)	0 (0)
	cT1G2	0 (0)	1 (6.5)
	cT1G3	3 [16]	1 (6.5)
	cT2	3 [16]	1 [7]
Concomitant Cis		3 [16]	0 (0)

Figures represent number of patients (% of tumors within the group) if not otherwise indicated

Cis: Carcinoma in situ; SD: Standard deviation

*One case was missing

**Missing cases are 3 in every group

### Omittable cystoscopies

The proportion of omittable cystoscopies was calculated for the entire group of patients with macroscopic hematuria (n = 2195). A primary cystoscopy could have been omitted in 57% (1260/2195) of the patients: those with a POS CTU (n = 174, representing 190 patients in the entire cohort), who would go to TURBT directly, and those with a NEG CTU (n = 77, representing 1070 patients in the entire cohort), (Additional file 1: Table 1). Accordingly, cystoscopy would have been done only in 43% (935/2195) of the patients: those who could not undergo CTU (n = 88, representing 456 patients in the entire cohort) and those with IND CTU (n = 46, representing 479 patients in the entire cohort). The number of cystoscopies needed to detect a false-negative CTU was thus 71 (1070/15), and for high-risk tumors the corresponding figure was 357 (1070/3).

## Discussion

Macroscopic hematuria is generally investigated with CTU and cystoscopy, the latter of which is an invasive procedure with patient discomfort and risk of complications. In the present study, we found that CTU with CMP had a low FNR and FPR, and cystoscopy might thereby be omitted in the majority of patients with macroscopic hematuria. Moreover, a high inter-observer agreement indicates that the results are valid in the clinical situation where there is only one radiologist interpreting the CTUs.

The low FNR and FPR found in this study are in close accordance with the prospective study by Helenius et al. who found sensitivity and specificity to be 0.87 and 0.99, respectively, when adding CMP to the normal CTU protocol [13]. However, their series included relatively few tumors, 55, compared to 207 in the present study which allows for a better estimate and analysis of false negative results. Also, these authors did not use a blinded revision of CTU and did not include a control group, in contrast to the present study.

In another retrospective study of 395 consecutive patients with macroscopic hematuria, CTU detected UBC in 13% of cases and with no indication of missed muscle invasive UBC [16]. In the present study, a negative CTU with good image quality and with no other bladder abnormalities missed tumors in only 15 of 207 (7%) patients with most of them low-risk tumors. Moreover, only three of these missed tumors were high-risk tumours, which accounted for 2.9% of high risk tumors group and 1.4% of all the patients with UBC. These tumors would have required a total of 1070 cystoscopies to detect.

In a mixed-methods study of patients’ preferences Tan et al. demonstrated that patients were willing to forgo cystoscopy if the sensitivity of the replacing biomarker was at least 0.90–0.95, recognizing that cystoscopy would also not detect all tumors [17]. This suggests that CTU with CMP should be interpreted for bladder tumors, not only upper tract tumors, and that cystoscopy could be omitted from the primary evaluation of macroscopic hematuria in case of a good-quality CTU with no pathological findings. In such a scenario, patients with a negative CTU who have recurrent hematuria would likely require cystoscopy which may detect the missed tumors, the extent of which will require further study. The consequences of missing high-risk tumors also need further investigation, and could potentially be mitigated by improvements in CTU protocols or the addition of urinary cytology and urinary biomarkers.

We found that cystoscopy could have been omitted in 57% of the hematuria investigations. To the best of our knowledge, no such analysis has been reported before. This is important since omitting cystoscopy may decrease the delay in diagnosis for cancer patients, avoid discomfort and complications of cystoscopy in the non-cancer cohort, and decrease health care costs [18]. This estimate is likely conservative, since it takes into account the 21% of patients who did not undergo CTU according to the protocol and such protocol might be improved if the bladder was a focus of the examination. One reason for including these patients in this estimate was to account for all patients that would be unable to undergo a full CTU, e.g., due to renal failure, which in this study was 14% (38/272) of the UBC group and 6% (7/113) of the control group.

In addition, the 22% of IND CTUs could likely be improved with increased experience in performing and interpretation of such CTU examinations and through modifications of the CTU protocol, especially with good filling of the urinary bladder before the examination. Possibly by further mobilizing the patient before taking the EP, we would obtain a more homogeneous contrast mixture in the bladder, which facilitates the detection of contrast defects. In addition, a higher diuresis using furosemide to ameliorate bladder filling might improve the CTU quality. We have accepted some noise in the images to keep the radiation dose as low as possible and it may be worth to consider whether we could have detected some of the smaller tumors if we had done the examinations with higher radiation dose in the arterial phase.

One concern in adding an additional contrast phase to the CTU protocol is the added radiation dose to the patients, however in this group of middle age and elderly patients, this is likely a minor concern. Another limitation with CTU in a world-wide perspective is its high costs and that not all hospitals or outpatient clinics have it. Furthermore, for practical reasons the study was not designed to evaluate whether other contrast phases could have been excluded. For example, excluding the EP or the NP would lower the radiation dose to the patients and take less time per patient, but the effects of such exclusions on tumor detection, especially in the upper tract, need further study.

Other modalities for bladder tumour detection such as biomarkers, ultrasound and magnetic resonance imaging are under development to improve the diagnosis of UBC [19–22]. Further studies are needed to elucidate whether these or CTU, either alone or in combination, can replace cystoscopy in diagnosing UBC.

The main strength of this study is that it is based on a large consecutive cohort of 2195 patients presenting with macroscopic hematuria examined with a standardized 4-phase CTU protocol and cystoscopy. An additional strength is that the CTUs were reviewed by two experienced radiologists who were blinded to each other and to all clinical data.

The primary limitation of the study is the retrospective design and that a relatively large proportion of the CTUs were not done according to the protocol. A further limitation is that not all negative CTUs were reviewed, instead a random sample of the negative controls was utilized. This was done to make the reviews of the CTUs manageable, while being able to maximize the number of patients with UBC and thereby estimate the main outcome, FNR, as close as possible. While this could potentially have led to an artificially low FNR by increasing the chance of interpreting the CTUs as positive, the very low FPR indicates that this did not happen. In addition, while we planned to include 100 patients as controls, the number of exclusions was larger than expected, but this did not affect the results since the FPR was much lower than expected.

As a conclusion, CTU with CMP can exclude UBC with high accuracy. For the majority of patients with a negative CTU it might be reasonable to omit cystoscopy, but prospective confirmative studies with possibly refined techniques and protocols for repeated hematuria are needed.

## Supplementary Information

**Additional file 1.** **Supplementary Table 1.** The results of the three groups 1 (POS, NEG and IND) and the group which did not undergo CTU in the study group (n = 297) and estimation for the entire cohort (n = 2195). The controls in the study group were multiplied by 17.02 (1923/113) to generalize to the entire hematuria group. Percentage of the column in parentheses. CTU: Computed tomography-urography.

## Data Availability

The data used to support the findings of this study are available from the corresponding author upon reasonable request.

## Abbreviations

CI: Confidence interval
CMP: Corticomedullary phase
CTU: Computed tomography urography
EP: Excretory phase
FNR: False negative rate
FPR: False positive rate
NP: Nephrographic phase
NPV: Negative predictive value
PPV: Positive predictive value
SD: Standard deviation
TNM: Tumor-node-metastases
TURBT: Transurethral resection of bladder tumor
UBC: Urinary bladder cancer
UP: Unenhanced phase
