# Supplementary material for: Computed tomography urography with corticomedullary phase can exclude urinary bladder cancer with high accuracy
Source: BMC Urol. 2022 Apr 12;22:60. doi: 10.1186/s12894-022-01009-4 (PMC9006563; doi:10.1186/s12894-022-01009-4)
Supplement: Supplementary file 1 — Additional file 1. Supplementary Table 1. The results of the three groups 1 (POS, NEG and IND) and the group which did not undergo CTU in the study group (n = 297) and estimation for the entire cohort (n = 2195). The controls in the study group were multiplied by 17.02 (1923/113) to generalize to the entire hematuria group. Percentage of the column in parentheses. CTU: Computed tomography-urography. [file 12894_2022_1009_MOESM1_ESM.pdf]

**Supplementary Table 1. The results of the three groups (POS, NEG and IND) and the group which did not undergo CTU in the study group (n=297) and estimation for the entire cohort (n=2195). The controls in the study group were multiplied by 17.02 (1923/113) to generalize to the entire hematuria group. Percentage of the column in parentheses.**

CTU: Computed tomography-urography.

| | **The study group** | | | **The study cohort** | | |
|---|---|---|---|---|---|---|
| | **Cancer** | **Control** | **Sum** | **Cancer** | **Control** | **Sum** |
| **CTU POS** | 173 (64) | 1 (1) | 174 (45) | 173 (64) | 17 (1) | 190 (9) |
| **CTU NEG** | 15 (6) | 62 (55) | 77 (20) | 15 (6) | 1055 (55) | 1070 (49) |
| **CTU IND** | 19 (7) | 27 (24) | 46 (12) | 19 (7) | 460 (24) | 479 (22) |
| **No CTU*** | 65 (24) | 23 (20) | 88 (23) | 65 (24) | 391 (20) | 456 (21) |
| **Sum** | 272 | 113 | 385 | 272 | 1923 | 2195 |

* Excluded from the study group
